# A collective case study of supervision and competence judgments on the inpatient internal medicine ward

**DOI:** 10.1007/s40037-021-00652-1

**Published:** 2021-01-25

**Authors:** Tristen Gilchrist, Rose Hatala, Andrea Gingerich

**Affiliations:** 1 Department of Medicine, University of British Columbia, Vancouver, Canada; 2 Centre for Health Education Scholarship, University of British Columbia, Vancouver, Canada; 3 Northern Medical Program, University of Northern British Columbia, Prince George, Canada

**Keywords:** Entrustment, Supervision, Competence, Assessment

## Abstract

**Introduction:**

Workplace-based assessment in competency-based medical education employs entrustment-supervision scales to suggest trainee competence. However, clinical supervision involves many factors and entrustment decision-making likely reflects more than trainee competence. We do not fully understand how a supervisor’s impression of trainee competence is reflected in their provision of clinical support. We must better understand this relationship to know whether documenting level of supervision truly reflects trainee competence.

**Methods:**

We undertook a collective case study of supervisor-trainee dyads consisting of attending internal medicine physicians and senior residents working on clinical teaching unit inpatient wards. We conducted field observations of typical daily activities and semi-structured interviews. Data was analysed within each dyad and compared across dyads to identify supervisory behaviours, what triggered the behaviours, and how they related to judgments of trainee competence.

**Results:**

Ten attending physician-senior resident dyads participated in the study. We identified eight distinct supervisory behaviours. The behaviours were enacted in response to trainee and non-trainee factors. Supervisory behaviours corresponded with varying assessments of trainee competence, even within a dyad. A change in the attending’s judgment of the resident’s competence did not always correspond with a change in subsequent observable supervisory behaviours.

**Discussion:**

There was no consistent relationship between a trigger for supervision, the judgment of trainee competence, and subsequent supervisory behaviour. This has direct implications for entrustment assessments tying competence to supervisory behaviours, because supervision is complex. Workplace-based assessments that capture narrative data including the rationale for supervisory behaviours may lead to deeper insights than numeric entrustment ratings.

**Supplementary Information:**

The online version of this article (10.1007/s40037-021-00652-1) contains supplementary material, which is available to authorized users.

## Introduction

With the development and implementation of competency-based medical education (CBME) and the concurrent emphasis on workplace-based assessment, entrustment rating scales are in the assessment spotlight [1]. Over the course of training, trainees are expected to progress towards increased autonomy with a decreased need for supervision, reflecting their readiness for independent clinical practice at the end of training [2]. The behaviourally anchored entrustment rating scales, initially developed in surgical contexts, include categories that describe varying amounts of supervision either based *retrospectively* on what the supervisor did do (i.e. moving from “I had to perform the procedure” to “I didn’t need to be there”) [3, 4] or *prospectively* making an inference about what the trainee could be allowed to do (i.e. “Presence allowed but no permission to enact EPA [entrustable professional activity]” to “May provide supervision to junior learners for this EPA”) [1]. While entrustment-supervision scales are designed to align with what supervisors do and capture a more holistic judgment of progression towards independence, rather than to be an assessment of competence [1], it can be argued that the implicit message of these scales conveys a judgment of competence. As such, this type of workplace-based assessment has woven together notions of entrustment, supervision, and competence with much remaining to be learned about how each informs the others.

The interweaving of entrustment, supervision, and competence can be examined as a starting point for understanding what the assessment information provided by documenting varying levels of supervision best represents. Entrustment decision-making, as described by ten Cate, can be conceptualized as distinct steps requiring progressively higher levels of inference, from observing performance, to judging competence, to making a judgment regarding entrustment and autonomy [1]. However, what appears to be a linear path towards an entrustment decision may actually represent a complex interaction of factors. Holzhausen’s conceptual model of entrustment decision-making [5] outlines how a trainee’s characteristics can lead to the intention to entrust, but the actual level of supervision provided is still moderated by perceived risk, supervisor characteristics, and the supervisor-trainee relationship. Consistent with this conceptualization, supervisors and trainees tend to place high levels of supervision and high levels of autonomy at opposite ends of a spectrum [6, 7]. Many factors nudge supervisors along the spectrum; the tension between allowing effective learning for the trainee and providing safe and effective care for the patient is the best described [7–9]. Furthermore, contextual factors such as available resources, competing tasks, time of day, and legal demands [5] may further influence the level of supervision provided. Thus, entrustment decision-making may not follow a single path from a judgment of competence to granting of entrustment.

Clinical supervision is a dynamic activity, including both a stable baseline supervisory style [8] and responsive supervisory behaviours [7, 9]. In their seminal work on clinical supervision, Kennedy et al.’s [7] observations in the emergency department and on the general medicine service revealed that while supervisors had a baseline level of clinical supervision (‘routine oversight’), they would increase this supervision (‘responsive oversight’) in response to trainee issues but also in response to* patient-specific* issues. With patient factors intertwined with myriad contextual factors and linked into entrustment decision-making and clinical supervision, there must be a complex relationship between the supervision that is provided to a trainee and the supervisor’s competence judgment of that trainee. The complexities of that relationship make it difficult to pinpoint the influence of non-trainee factors on the information documented with entrustment-supervision scales. While Kennedy’s work highlights changes in supervision behaviours and some of the factors influencing these changes, there is no explicit link between the supervision provided and the supervisor’s competence judgment of the trainee. Understanding this relationship is crucial if we are to employ assessment tools that document levels of supervision.

In the current study, we aim to follow a supervisor’s judgment of competence, their supervisory behaviours, and their entrustment decision and extend Kennedy’s work to address the following questions: What informs the level of supervision provided to a trainee for a specific task, and how do the levels of supervision align with judgments of trainee competence?

## Methods

### Design

Drawing inspiration from Kennedy’s method of using observational fieldwork and brief interviews to study clinical supervision [7], assessment of trainees’ competence for independent clinical work [6] and help-seeking by trainees [10], we observed the interactions between supervisor-trainee dyads on inpatient wards. In contrast to the grounded theory methodology used by Kennedy and her team, we employed case-study methodology, which “facilitates exploration of a phenomenon within its context” [11] and is a design that is well-suited to research in complex settings where concepts may not have simple causal relationships [12]. A collective case study combines a set of cases, wherein each case is less important than gaining an understanding of the theoretical underpinnings of a unit of analysis within the cases [11, 13]. We defined each case as one attending-resident dyad who were randomly scheduled by the residency program to work together for a two-week period. We approached the cases using an interpretivist paradigm—taking reality as a social construct arising from how individuals experience the world and interact with one another—which allows for the exploration and deeper understanding of a particular concept within and across the cases while considering the importance of environmental context [12].

### Educational setting

The study took place on the internal medicine clinical teaching unit (CTU) inpatient wards at three University of British Columbia (UBC)-associated tertiary care hospitals.

### Participants

Chief medical residents sent invitations to senior medical residents and attendings scheduled at the three CTU sites. Recruitment targeted attendings who had earned a reputation among their supervisor colleagues (TG, RH) on the research team for ‘empowerment’ and ‘mixed practice’ styles [8] because supervisors using these styles are more likely to adjust their level of supervision based on an assessment of the trainee’s competence than those using ‘minimalist’ and ‘direct care’ styles with unchanging levels of supervision. Attending participants included general internists or geriatricians on the internal medicine service, with experience spanning from a pre-certification junior attending to more than 15 years in practice. Trainee participants included second- and third-year internal medicine residents spanning their first to last rotation as a senior resident. All dyads were unique with residents and attendings recruited to participate in a single dyad to maximize variation in sampling.

### Data collection

We collected data through field observations and semi-structured interviews. Each dyad was observed for up to seven hours per day on two days approximately one week apart. Observations focussed on the senior resident and their typical daily activities on the ward in order to note the resident’s apparent or voiced need for support, the presence or absence of the attending physician, and the interactions between the resident and the attending. The activities included rounding on patients, overseeing the work of junior trainees (medical students and junior residents), meeting with allied health representatives (‘team care’), teaching junior trainees, and reviewing with the attending physician the care decisions for all patients assigned to the team (‘running the list’). Observations were documented in field notes. At the end of the workday, each member of the dyad participated in a separate semi-structured interview. Both were prompted to discuss incidents observed during the day in which attending physicians exhibited supervisory behaviours or might have been expected to engage in supervisory behaviours. Other questions related to the resident’s need for support, and the competence judgments of the attending. Within a few weeks after the second observation day, there was an additional semi-structured interview with the attending physician to discuss any further developments in their work with the senior resident and to review their final assessment of the resident. Interviews were audio-recorded, transcribed, and de-identified.

### Researcher roles and backgrounds

At the beginning of the research process, TG was a final-year general internal medicine resident in supervised practice. Later in the process, TG was working as a general internist and was at times supervising residents and medical students. TG had a pre-existing relationship with some residents in the study and with all of the attending physicians. AG is a PhD education researcher with expertise in rater cognition. RH is an internal medicine and palliative care physician and education researcher with expertise in feedback and assessment. Our use of an interpretivist paradigm made it important to engage in the process of reflexivity [14]. We aimed to continually maintain consideration of how researcher perspectives and biases could be influencing interpretation of the data [15] by engaging in frequent research meetings, during which we explored how our unique backgrounds influenced our understanding and analysis.

### Data analysis

Following collective case study methodology [11, 16], we engaged in a recursive process of examining data to simultaneously summarize and interpret it during its collection to inform modifications to subsequent observations, field notes, and interview questions. Once collected, we analysed the data within each case and then compared across cases. Within-case analysis began with all three researchers independently analysing each dyad’s interview transcripts and field notes to identify incidents involving supervisory behaviours (e.g. attending goes to bedside with resident or attending does not go to team rounds) or incidents in which we might have expected a particular supervisory behaviour (e.g. patient suddenly deteriorates or resident struggles at team rounds). These were discussed in a group meeting, and then one researcher (AG) returned to the data and verified these incidents for accuracy and consistency, compiling all available information from field notes and interview transcripts that pertained to that incident. Through further review and discussion of the compiled information, the research team developed a systematic procedure [13] to organize the information pertaining to each incident by parsing it into information that described the supervisory behaviour, the trigger of the supervisory behaviour, why the attending responded to that particular situation with that particular supervisory behaviour, how the incident informed their judgment of trainee competence, and any impact on subsequent supervision. Such organization enabled the sequence of incidents to be tracked within each dyad and facilitated our examination for any pattern of changes within each dyad, particularly in regard to judgments of the resident’s competence.

For the cross-case analysis, the organization of the incident information allowed for it to be re-sorted to focus on each supervisory behaviour rather than on each dyad. This enabled each supervisory behaviour (e.g. goes to bedside with trainee) to be tracked across incidents in multiple dyads and facilitated our examination of the information for any patterns, in particular the alignment with competence judgments for various residents.

### Ethics

Ethics approval was received from the University of British Columbia behavioural research ethics board (H18-00878). Appropriate institutional approvals were also obtained from the health authorities (Fraser Health, Providence Health Care, and Vancouver Coastal Health) where the observations took place.

## Results

Ten attending physician-senior resident dyads participated in the study between October 2018 and August 2019, providing 10 cases for analysis. Due to logistical issues, one dyad was a triad consisting of a senior resident, junior attending physician, and senior attending physician. As is usual in this setting, the team of learners (including the senior resident) treated the junior attending as if they were the attending, while the actual attending physician was largely absent from the wards. All three were interviewed twice. Four dyads were observed and interviewed by the lead of the research team (TG), three dyads were observed and interviewed by both TG and a research assistant as part of training, and three dyads were observed and interviewed only by the research assistant. This produced 51 interview transcripts and 25 sets of daily field notes. Analysis within each case identified 1–7 supervisory incidents per case for a total of 37 incidents. To protect the identity of the dyads, we have removed specific details of the incidents from the quotes and converted gendered pronouns to gender neutral terms or to participant numbers (where “A” refers to an attending physician and “R” refers to a senior resident trainee).

### Within-case analysis: supervisory behaviours identified

We identified eight supervisory behaviours, which are listed in Table 1 (found in the Electronic Supplementary Material [ESM]) with representative examples, through examination of the incidents. The incidents were labelled by the supervisory behaviour that represented a change or a choice in the level of supervision provided to the resident at that moment. More specifically, the supervisory behaviour ‘providing direct patient care’ refers to incidents where the attending delivered care for the patient themselves with the resident present (dyad 63) or not (dyads 15, 20). The supervisory behaviour ‘going to the bedside’ represents an attending’s decision to accompany the resident as they provide patient care (dyads 3, 18, 41, 72, 98). The supervisory behaviour ‘helping to navigate hospital logistics and bureaucracy’ (dyads 15, 24, 41) reflects incidents where the attending led administrative tasks to procure the patient’s access to investigations or resources. The supervisory behaviour ‘taking over the lead’ during group discussions refers to the attending stepping into a leadership role during team care meetings or running the list discussions (dyads 15, 16, 20, 41, 98). The label ‘interjecting to change treatment or management’ (dyad 3) refers to the attending correcting the resident’s orders whereas the label ‘answering trainee questions and helping with decision-making’ (dyads 3, 15, 41, 72) reflects a more supportive discussion of how the resident could proceed. The attending providing a more substantial teaching moment inspired by the immediate incident but focusing on future responsibilities was labelled ‘providing directed teaching’ to the trainee (dyad 63). The supervisory behaviour ‘checking up and checking behind’ referred to the attending monitoring the resident’s performed activities without directly observing them (dyads 3, 16, 20, 63, 98) or while physically present but not directly involved with the activities (dyad 16).

### Within-case analysis: supervisory behaviours triggered by trainee and non-trainee factors

Supervisory behaviours were used in response to factors related to the resident and also for factors unrelated to the residents. The supervisory behaviour ‘going to the bedside’, for example, was used in response to trainee factors, such as their perceived ability, observed performance, and experience with the task. It was used to support the resident when support was needed (dyad 18), to assist the resident after they had asked for help (dyad 72), and as a means to identify a performance gap and then provide feedback to the resident (dyad 98). However, the supervisory behaviour ‘going to the bedside’ was also used when the clinical situation (i.e. non-trainee factors) warranted, such as when the patient was acutely sick or dying (dyads 18, 98), as a means of checking in with nursing staff (dyad 41), and as a routine part of supervision with “zero concern with the [resident]” (dyad 18) and when the resident did not need or ask for support (dyads 15, 98, 41).

### Within-case analysis: competence judgments informing supervisory behaviours

Supervisory behaviours were not always informed by judgments of a resident’s competence. When they were, the choice to use a given supervisory behaviour, such as ‘checking up and checking behind’, could be informed by either previous impressive performance (dyads 3, 16, 63) or unimpressive performance (dyad 98). A supervisory behaviour informed by a competence judgment did not necessarily result in the resident receiving the level of supervision they needed, and this could be realized at a later time. For example, “R3 strikes me so far as being capable in terms of a manager, in terms of being sufficiently assertive and able to direct team care” so the attending began to arrive late at team care and then stay in the background before ultimately not attending team care or a running the list session. Although the resident was eventually allowed to do the task without the attending present, when the attending ‘checked up and checked behind’, A3 discovered “a couple days later that maybe there were things that could have been done better”, revealing that the resident did not perform as competently as expected and could have used supervisory support during team care.

A change in the attending’s judgment of the resident’s competence did not always correspond with a change in subsequent supervisory behaviours. In one incident, the attending discovered that R20 had delayed important care while working solo overnight. The attending acknowledged: “I think that there’s some knowledge gaps in the way [the resident] manages things” and that “maybe someone stronger probably would have known”. Thus, the attending’s judgment of the resident’s competence was that “maybe R20’s not quite as strong as [I thought] R20 was before”. However, when asked directly if they changed how they supervised the resident after the incident, A20 clearly responded “no”, and no change was observed.

### Within-case analysis: patterns of supervisory behaviours aligning with competence judgments

The supervisory behaviour used in an incident was not an obvious indication of the attending’s judgment of the resident’s competence in that incident. For example, a supervisory behaviour that indicates less resident autonomy, like ‘answering trainee questions and helping with decision-making’, could align with an *increased *judgment of competence as happened when R3 could not identify a medical condition indicated by laboratory results and asked for help. Despite the attending acknowledging “a minor knowledge gap in terms of maybe not necessarily knowing this disease entity and what it signified”, their judgment of the resident’s competence slightly increased because “R3 knew enough of that pattern being abnormal that it required more attention which I thought was reassuring for somebody at their level of training.”

Similarly, the pattern of supervisory behaviours used within a dyad was not a good indication of the attending’s overall judgment of the resident’s competence. For example, the five incidents of dyad 15 seem to paint a picture of a trainee who repeatedly needed the attending to step in and provide support: the attending ‘took over the lead’ during team care and then 20 minutes later when the resident asked about how to manage a patient’s fitness to drive, the attending ‘helped to navigate bureaucracy’ by taking it on themselves before offering to ‘provide direct patient care’ for other patients while the resident was off the ward for teaching. The next week the attending ‘provided direct patient care’ to a patient with a life-threatening condition that the resident could not handle in the morning and then ‘helped the trainee with decision-making’ for a patient with a rare condition in the afternoon. However, the attending described the resident as “a very strong clinician” and when asked why they used these supervisory behaviours, they said they were helping “in anticipation of the weekend and the limited services available” and for “guidance about how to deal with this quite complicated issue because it’s something that [the resident] may not have seen before.”

### Cross-case analysis: supervisory behaviour as an indication of judged competence

When the identified supervisory behaviours were tracked through numerous incidents across multiple dyads, they corresponded with varying assessments of resident competence. For example, the supervisory behaviour ‘answering trainee questions and helping with decision-making’ was used when the resident’s competence with that task ranged from “maybe a minor knowledge gap” (A3) to “understanding was there for R72 but they just maybe lacked the confidence to make that decision on their own” (A72) to “R41 actually is highly detail oriented and keeps excellent notes and knows what’s going on with the patient” (A41). The supervisory behaviours that involved the attending stepping in to provide additional support tended to correspond with them saying that their judgment of resident competence was unaffected or even high for that task. They made statements like this was “a very strong [resident]” (A63) and “easily a task I could have completely delegated to the [resident] to do” (A41) but regardless they stepped in for non-trainee factors such as having the time to help (dyad 15) or to increase efficiency on ward (dyad 41) or it was a very sick patient or unusual diagnosis (dyads 98, 15) or they just happened to be close-by and “thought it could have been a terminal event and I should be there as the attending physician” (A18).

## Discussion

In this study, we observed and interviewed supervisor-trainee dyads as they engaged in their daily clinical work on the internal medicine inpatient ward. We focussed on noting the triggers for supervision and the responsive supervisory behaviours, and explored the associated underlying supervisor judgment of trainee competence. Within dyads, we found that the supervisor’s judgment of trainee competence was relatively unchanging while their supervisory behaviour varied from providing low to high levels of support in different incidents. Conversely, any given supervisory behaviour was associated with varied competence judgments when tracked across different dyads. Both within dyads and across dyads, there was not a consistent relationship between the trigger for supervision, the supervisor’s competence judgment of the trainee, and the supervisory behaviour.

Our findings contribute to the current conceptualizations of the relationship between supervision, competence, and entrustment decision-making. Both Cianciolo and Holzhausen’s [5, 17] conceptual models of entrustment decision-making have highlighted the importance of contextual factors on the entrustment decision. In Holzhausen’s conceptual framework [5] these contextual factors moderate the process between the supervisor being ready to entrust and the supervisor’s enacted level of supervision. This influence of non-trainee factors on supervisory behaviours may help explain our findings that supervisory behaviours are not always related to the trainee or their actions and that the level of support provided does not have a consistent relationship with the supervisor’s competence judgment.

Our analysis, grounded in case-study methodology, reveals that within a dyad, supervisors move fluidly between increasing and decreasing the support provided to the trainee in response to both trainee factors (such as a request for help or their inexperience with the task, but not typically their perceived competence) and non-trainee factors (i.e. patient or clinical context). It was common for the supervisors to provide increased support in response to non-trainee factors, suggesting that patient and contextual factors play an important role in triggering the level of supervision provided to trainees. While all of the previous models of clinical supervision in internal medicine [7–9, 18, 19] described both trainee and non-trainee factors that influenced supervisory behaviours, our results underscore that non-trainee factors are at least as important as trainee factors, reflecting the supervisor’s dual roles as patient care provider and clinical educator. When considering the tension between patient care and clinical education, the patient care axis may even be the more dominant driver of supervisory behaviour on the inpatient ward.

Our results have direct implications for assessments based on entrustment decision-making, as envisioned in contemporary CBME. In our Canadian context, the recommended entrustment-supervision assessment tool is the O‑SCORE, which uses the retrospective scale of documenting the supervisor’s behaviour in a recent moment with respect to a specific entrustable professional activity (EPA) [3, 4]. Implicit within these retrospective ratings is a relationship between a supervisor’s judgment of a trainee’s competence and the amount of autonomy the trainee is granted, as reflected in the level of supervision provided. In this assessment paradigm, multiple independent observations and assessments of EPAs are meant to provide a picture of trainee competence. However, our results highlight a significant challenge to these ratings for inpatient, ward-based medicine, in that the amount of supervision provided is often due to factors other than the trainee and does not appear to be well-aligned with the competence judgment of that trainee. In our dyads, using a model of retrospective assessment based on supervisory behaviour would likely have misrepresented the judgment of trainee competence. Looking at the supervisory behavior ‘checking up and checking behind’, an attending could have different competence judgments of two residents, but ultimately select “I didn’t have to be there” on an entrustment rating scale based on the level of supervision described by that behaviour. This finding is problematic for entrustment-based assessments that imply a direct link between supervision provided and judgment of competence.

For assessment purposes, what may be more informative than capturing supervisory behaviour on a rating scale is capturing the explanation and rationale for that behaviour. The explanations shared by supervisors in this study for why they provided a certain level of support to a trainee and the explicit expression of their assessment of the trainee’s competence were very informative. For example, explicating the rationale for two different supervisors’ ‘checking up and checking behind’ behaviour and their underlying competence judgments would lead to a very different understanding of the clinical performance of their respective trainees. This highlights that capturing narrative data in our workplace-based assessments may lead to deeper insights regarding trainees and supervisors than a numeric entrustment rating provides [20–22].

Our results replicate many of the seminal findings of Kennedy et al. [7]. We found that supervisors demonstrate a continuum of levels of support ranging from routine oversight, which is done irrespective of the trainee’s competence, to responsive oversight and on to provision of direct patient care, which are often invoked in response to external factors. We also confirmed evidence of backstage oversight (the supervisory behaviour of ‘checking up and checking behind’) occurring unbeknownst to the trainee. Our study extends Kennedy’s findings by following the supervision temporally forward (from trigger to response) and explicitly exploring the associated judgment of trainee competence. We found that some expected triggers for supervision do not provoke increased support, such as trainees asking questions that do not impact competence judgments. Conversely, many instances of providing increased levels of support do not reflect a change or downgrade in a judgment of trainee competence.

The uniqueness of our study is that we were able to follow a trigger for supervision through to the supervisory action and the associated competence judgment. However, there are limitations to our study. Our participants were volunteers, and our sample likely skews towards competent and highly competent trainees. We thus have less insight into the relationship between supervision and competence judgment for lower-performing trainees. The study is located in one university’s residency training program, at three urban tertiary care hospital sites, which may limit transferability of these results to other training contexts. Our observations were limited to two days of a 14-day dyadic working relationship, and thus we may not have observed the full range of triggers and supervisory behaviours. Our trainees were residents in their second and third year of a minimum 4‑year training program, so there was no expectation of full autonomy or entrustment of all aspects of patient care. Finally, by observing each attending with only one resident and by following the resident rather than the attending, we were unable to see how an individual attending adjusts to different residents and may have missed some of their backstage oversight activities (though we explored this within the interviews).

Our study points to a number of ways supervision and entrustment could be further unpacked in future research. Observing and interviewing dyads at the start of their working relationship (as opposed to randomly during their rotation together) would allow exploration of the development of the supervisor’s initial competence judgment. Including a broader spectrum of trainee performance and years of training would facilitate examining whether and how the supervisory behaviours change in response to lower trainee competence or to trainees nearing the end of training. Including a single supervisor with different trainees would allow for exploration of how supervisory behaviours within an individual may or may not change based on the trainee. Examining actual supervision in contexts where independent practice is possible (such as general practitioner training in the Netherlands) and where prospective entrustment ratings are employed could yield further insights into the relationship between competence, supervision, and autonomy.

What is apparent is that there is not a single path to follow that links a trainee’s performance to the supervisor’s judgment of that trainee’s competence and the supervisor’s provision of support, but rather a complex and varied interweaving of multiple factors. We must remind ourselves of this complexity as we continue to implement assessments based on notions of entrustment and levels of supervision.

## Supplementary Information

**Table 1** Observed supervisory behaviours with representative examples illustrating the trigger for the behaviour and the corresponding judgment of the trainee’s competence.
