# Supplementary material for: A collective case study of supervision and competence judgments on the inpatient internal medicine ward
Source: Perspect Med Educ. 2021 Jan 25;10(3):155–62. doi: 10.1007/s40037-021-00652-1 (PMC8187503; doi:10.1007/s40037-021-00652-1)
Supplement: Supplementary file 1 — Table 1 Observed supervisory behaviours with representative examples illustrating the trigger for the behaviour and the corresponding judgment of the trainee’s competence. [file 40037_2021_652_MOESM1_ESM.docx]

**Table 1** Observed supervisory behaviours with representative examples illustrating the trigger for the behaviour and the corresponding judgment of the trainee’s competence. ‘A’ refers to an attending physician while ‘R’ refers to a resident physician

| **Specific example** | **Triggering incident** | **Corresponding competence judgment** |
| --- | --- | --- |
| Supervisory behaviour: *attending taking the lead in* ‘*providing direct patient care’ with or without the resident present* | | |
| “A15 was managing the patient [with the difficult hospital disposition], so A15 just took care of it and we were in contact throughout. So I knew what A15’s plan was.” (R15) | “This is really important; I don’t want to skate by this too quickly because this is potentially life-threatening.” (A15 quoted in field note)  “In anticipation of the weekend and the limited services available over the weekend. Trying to coordinate all those services is also a bit stressful and challenging.” (A15) | “R15 is a very strong clinician, but R15 also fostered a very positive environment with the rest of the team.” (A15) |
| Supervisory behaviour: *attending is* ‘*going to the bedside’ with the resident as they provide patient care* | | |
| "In the hallway when [the patient] was kind of flailing around […] we both kind of co-managed the situation" (R18) | "Oh, no, I just overheard [the commotion] – because I was charting I guess five metres away and then I heard R18’s voice." (A18) | “I would have done the same thing as R18 did and R18 was verbalizing all the plans which I do – did agree with.” (A18) |
| Resident going to see a couple of patients who will be discharged today. One patient didn’t want to go home. (Dyad 72 field notes) | “I didn’t ask for, I guess, help directly, but I brought the case to my attending, kind of talked over the barriers and challenges that were being brought up. A72 offered to chat with the patient as well to kind of provide a different perspective or to try to, I guess, like, not consolidate but to also reaffirm what I had started with the patient. So I think it was a bit of providing backup in that case.” (R72) | “I think that R72’s doing very well. So I don’t have to provide them with that much support, but – I think more just reassurance, like, R72’s lacking confidence, is how I supported R72 mostly. And just me having the benefit of a bit more experience and knowledge.” (A72) |
| Supervisory behaviour: *attending* ‘*helping to navigate hospital logistics and bureaucracy’ by leading administrative tasks to procure investigations or resources for the patient* | | |
| A24 responded by asking R24 to meet A24 in ICU and ask for ICU’s help together. A24 encouraged R24 to request support from ICU with A24 present to back R24 up, only weighing in with A24’s agreement that they needed to have help and an outside opinion. (Dyad 24 field notes) | “When we were making the decision whether or not to involve ICU or not in that patient, I knew A24 was in a meeting. […] I really wanted A24’s opinion, what do you think we should do? Should we call ICU? Should we do this?” (R24) | “I thought all the interactions were appropriate, professional. R24 showed foresight and consideration and – R24’s efficient […] R24 has good judgment and R24 knows their limitations. I try to create an environment where they can come to me ask me ‘what do you think?’” (A24) |
| Supervisory behaviour: *attending* ‘*taking over the lead’ by stepping into a leadership role during team care meetings or running the list discussions* | | |
| A98 takes over directing team care for protocol and safety issues. (Dyad 98 field notes) | "Trying to get some sort of cohesive plan for some of these patients has been challenging. And so I did take over maybe a little bit more even specifically today even. Just because the [allied health team member] doesn’t even understand stuff. And the-- and some of the other team members are a little frustrating to me. So I was being a bit more directive." (A98) | “I take the philosophy, especially when they’re early [like R98 is], that I think the nuances of discharging these complex elderly people is a bit too much for them and I take over. I hope that’s going to be okay with R98.” (A98) |
| A20 steps in to redirect and reorganize investigation with junior resident so R20 steps back during running the list discussion. (Dyad 20 field notes) | “I pressed [the junior resident] on that and then in the pressing her on that I got confused. I got caught up in my own thoughts about what [the appropriate treatment] would be. And then A20 jumped in and corrected me and then I was trying to correct myself. […] I’m glad A20 clarified when they did because I was saying the wrong thing." (R20) | “We’ve had moments like that where R20 didn’t know something or whatever. So I think it makes R20 realize that R20 doesn’t know maybe as much as R20 should know. But it’s more that R20’s going to be harder on themself rather than anything else, I think. Because R20 has enough insight to know this kind of stuff [...] R20's reasonably thorough." (A20) |
| Supervisory behaviour: *attending* ‘*interjecting to change treatment or management’ and correct the resident’s orders* | | |
| Medical student poses a treatment suggestion. Resident agrees. Attending had been working on computer: disagrees with resident, briefly explains why, describes opposite treatment approach. (Dyad 3 field notes) | Nurse comes in and says patient they’re currently talking about looks really sick. (Dyad 3 field notes) | “I think R3 strikes me so far as being capable.” (A3) |
| Supervisory behaviour: *attending* ‘*answering trainee questions and helping with decision-making’ as needed to support the resident with providing care* | | |
| R41 questions A41: “[The patient’s] urine grew a culture so I don’t know if they should treat it …” (Dyad 41 field notes) | “The issue around whether or not to treat someone’s urinary infection that – it was more because A41 was there after team care that we chatted about, I think that particular scenario, there wasn’t really a right or a wrong and the patient was very stable. So it was – there was time to think about the different courses of action and what would be the next step depending on the outcome of that. Yeah, I appreciated bouncing ideas off of A41.” (R41) | “Even in these four days I’ve gotten to know the senior resident much better, and understand that R41 actually is highly detail oriented and keeps excellent notes and knows what’s going on with the patient.” (A41) |
| Supervisory behaviour: *attending* ‘*providing directed teaching’ to the resident inspired by the current incident but intended to focus on future responsibilities* | | |
| A63 asking R63: “What would you do if a resident was presenting a case and was doing a bad job? … if there were several issues?”  R63 shares their approach to the hypothetical situation before A63 discusses alternate strategies for giving constructive feedback.  (Dyad 63 field notes) | “I haven’t [seen R63 struggle with giving constructive feedback], but I think it was more just based on my perception that it’s an area that we don’t spend probably enough time thinking and talking about it. Something that we’re kind of just expected to know how to do, but that we don’t necessarily have any specific training in.” (A63) | “I think R63’s got a good foundation around the core competencies in terms of medical knowledge, application of medical knowledge. And so I wanted to just probe and also provide R63 with a bit of an opportunity to think about some of the other skills that we need as physicians and as educators in terms of how we assess trainees. And also how we deliver constructive but sometimes difficult feedback to people that we’re supervising.” (A63) |
| Supervisory behaviour: *attending* ‘*checking up and checking behind’ to monitor the resident’s activities without directly observing them or being directly involved with them* | | |
| A16 was not there for the morning meeting.  R16 texts A16: “I’ll let the boss know that we divided the list.”  A16 on the unit. R16 is talking to her about the patient R16 just saw.  A16 leaves and says, “I’ll be back.”  A16 told R16 they’ll meet later to discuss a few things. (Dyad 16 field notes) | “It’s nice to have independence and it’s nice to be able to practice some of those skills, but also to have the support when you need it […] Today felt a little bit different from the standpoint of I think A16 had decided in advance not to [be hands-on].” (R16) | “Today was really just the [trainee] running the show and I’m just in the backdrop. […] We formulate game plans and it’s really just carrying it out. But also giving the autonomy to teach and run the list and do everything that – R16’s obviously very adequately skilled to do. So trying not to interfere is really my goal for today.” (A16) |
